# Gastrointestinal Manifestations in Systemic Mastocytosis: The Need of a Multidisciplinary Approach

**DOI:** 10.3390/cancers13133316

**Published:** 2021-07-01

**Authors:** Magda Zanelli, Marco Pizzi, Francesca Sanguedolce, Maurizio Zizzo, Andrea Palicelli, Alessandra Soriano, Alessandra Bisagni, Giovanni Martino, Cecilia Caprera, Marina Moretti, Francesco Masia, Loredana De Marco, Elisabetta Froio, Moira Foroni, Giuditta Bernardelli, Maria Isabel Alvarez de Celis, Alessandro Giunta, Francesco Merli, Stefano Ascani

**Affiliations:** 1 Pathology Unit, Azienda USL-IRCCS di Reggio Emilia, 42123 Reggio Emilia, Italy; andrea.palicelli@ausl.re.it (A.P.); alessandra.bisagni@ausl.re.it (A.B.); loredana.demarco@ausl.re.it (L.D.M.); elisabetta.froio@ausl.re.it (E.F.); moira.foroni@ausl.re.it (M.F.); giuditta.bernardelli@ausl.re.it (G.B.); 2 General Pathology and Cytopathology Unit, Department of Medicine-DMED, University of Padova, 35121 Padova, Italy; marco.pizzi.1@unipd.it; 3 Pathology Unit, Policlinico Riuniti, University of Foggia, 71122 Foggia, Italy; francesca.sanguedolce@unifg.it; 4 Surgical Oncology Unit, Azienda USL-IRCCS di Reggio Emilia, 42123 Reggio Emilia, Italy; maurizio.zizzo@ausl.re.it (M.Z.); alessandro.giunta@ausl.re.it (A.G.); 5 Clinical and Experimental Medicine PhD Program, University of Modena and Reggio Emilia, 41121 Modena, Italy; 6 Department of Pathology, Case Western Reserve University, Cleveland, OH 44106, USA; alessandra.soriano@ausl.re.it; 7 Gastroenterology Division, Azienda USL-IRCCS di Reggio Emilia, 42123 Reggio Emilia, Italy; 8 Pathology Unit, Azienda Ospedaliera Santa Maria di Terni, University of Perugia, 05100 Terni, Italy; gio.martino@gmail.com (G.M.); ceciliacaprera@libero.it (C.C.); s.ascani@aospterni.it (S.A.); 9 OncoHematology Unit, Azienda Ospedaliera Santa Maria di Terni, University of Perugia, 05100 Terni, Italy; m.moretti@aospterni.it (M.M.); f.masia@aospterni.it (F.M.); 10 Hematology Unit, Azienda USL-IRCCS di Reggio Emilia, 42123 Reggio Emilia, Italy; alvarezdecelis.mariaisabel@ausl.re.it (M.I.A.d.C.); francesco.merli@ausl.re.it (F.M.); 11 Haematopathology Unit, CREO, Azienda Ospedaliera di Perugia, University of Perugia, 06129 Perugia, Italy

**Keywords:** mast cell, mastocytosis, mast cell activation, bone marrow, gut

## Abstract

**Simple Summary:**

Mastocytosis is a group of neoplastic mast cell disorders ranging from a skin-limited disease to a systemic form with multi-organ involvement, including gut involvement. Clinical manifestations and outcome of systemic mastocytosis are variable. Symptoms may result from either release of mast cell mediators or tissue infiltration by mast cell proliferation. Gastrointestinal symptoms are one of the major causes of morbidity in these patients. The diagnosis of gastrointestinal mastocytosis can be tricky, as symptoms often mimic other more common gastrointestinal diseases; the endoscopic appearance is often unremarkable or nonspecific and the infiltrate can be focal and subtle and easily missed unless special stains are used. This review aims to better define the gastrointestinal involvement in systemic mastocytosis, discussing potential diagnostic pitfalls and pointing out the importance of a multidisciplinary approach for a prompt diagnosis and treatment.

**Abstract:**

Mastocytosis represents a heterogeneous group of neoplastic mast cell disorders. The basic classification into a skin-limited disease and a systemic form with multi-organ involvement remains valid. Systemic mastocytosis is a disease often hard to diagnose, characterized by different symptoms originating from either the release of mast cell mediators or organ damage due to mast cell infiltration. Gastrointestinal symptoms represent one of the major causes of morbidity, being present in 60–80% of patients. A high index of suspicion by clinicians and pathologists is required to reach the diagnosis. Gastrointestinal mastocytosis can be a challenging diagnosis, as symptoms simulate other more common gastrointestinal diseases. The endoscopic appearance is generally unremarkable or nonspecific and gastrointestinal mast cell infiltration can be focal and subtle, requiring an adequate sampling with multiple biopsies by the endoscopists. Special stains, such as CD117, tryptase, and CD25, should be performed in order not to miss the gastrointestinal mast cell infiltrate. A proper patient’s workup requires a multidisciplinary approach including gastroenterologists, endoscopists, hematologists, oncologists, and pathologists. The aim of this review is to analyze the clinicopathological features of gastrointestinal involvement in systemic mastocytosis, focusing on the relevance of a multidisciplinary approach.

## 1. Introduction

Mast cells (MCs) are multifunctional cells involved in innate and acquired immunity and attendant inflammatory reactions [1,2,3]. They have high-affinity receptors for IgE (IgERs) and synthesize inflammatory and vasoactive mediators, which are stored in the metachromatic granules of mature MCs [1,2,3]. After activation, MCs determine clinical manifestations through mediator release [1,2,3].

Mast cell activation (MCA) may occur in different physiologic and pathologic conditions [3]. Clinical symptoms resulting from MCA can be observed not only in the setting of allergic diseases, but even in the context of MC neoplasms [3]. If MCA symptoms are severe and recurrent, the possibility of mast cell activation syndrome (MCAS) should be considered [3,4].

Mastocytosis represents a highly heterogeneous group of neoplastic MC disorders, characterized by abnormal growth and accumulation of MCs in one or more organ system [5]. Its clinical presentation is variable with a course ranging from indolent to aggressive [5].

The basic classification of mastocytosis into pure cutaneous forms (90%) and systemic forms (10%) remains valid. The pure cutaneous forms are mainly pediatric with often spontaneous regression at puberty and a favorable outcome [5]. Multi-organ involvement, with or without a cutaneous disease, is generally seen in adult patients, with the most advanced forms of systemic mastocytosis (SM) usually lacking skin involvement. The diagnosis of SM in absence of skin involvement, may be particularly challenging and needs a high index of suspicion. In SM, the most commonly involved sites are bone marrow (BM), liver, spleen, gastrointestinal tract (GIT), and lymph nodes [5]. Symptoms can result from either release of MC mediators or organ damage due to MC infiltration.

Gastrointestinal (GI) symptoms are present in 60–80% of SM patients, representing one of the major causes of morbidity [6,7,8,9]. GI symptoms are largely caused by release of mediators and in rare advanced forms by MC infiltration of the gut causing malabsorption. Direct gut involvement by neoplastic MCs has been documented only in a limited number of cases and the histopathologic spectrum of GI mastocytosis is still incompletely characterized [6,7,10]. Because of its multifaceted manifestations and progression, mastocytosis is a disease hard to diagnose, with different specialists involved in the patient’s clinical work-up. An early and prompt diagnosis is of importance not only to cure disabling symptoms, but also to limit disease progression.

## 2. Mast Cells and Mast Cell-Related Disorders

### 2.1. Mast Cell Physiology

MCs develop from pluripotent precursors in BM; they circulate in the blood as MC precursors and differentiate after migration into different tissues, including skin, lungs, and GIT, in response to tissue-specific cytokines [11]. Mature MCs contain granules storing mediators such as histamine, enzymes and neutral proteases. The majority of the granule components is made by neutral proteases, including chymase and tryptase [11].

MCs are activated on binding to an antigen, which cross-links antigen-specific IgE on the MC surface [11]. Non-specific triggers such as the physical stimuli of pressure and stress and some substances (alcohol or drugs) may also cause MCA and subsequent secretion of vasoactive and proinflammatory mediators. GI symptoms have been specifically attributed to mediators including histamine, platelet activating factor (PAF), prostaglandin D2 (PGD2), serotonin, tryptase, leukotrienes, tumor necrosis factor-alfa (TNF-alfa), and interleukin-6 (IL-6) [11,12]. Normally, MCs in the gut account for 2–5% of mononuclear cells in the lamina propria [8,13]. MCs are usually scattered in the gut mucosa of healthy subjects; in irritable bowel syndrome (IBS) patients, MCs may be increased in number, but are dispersed without forming aggregates, whereas MCs aggregates may be observed in GI mucosa of SM patients [13].

### 2.2. Mast Cell Activation Syndrome: Consensus Criteria and Classification

MCA may occur in different physiologic and pathologic conditions. In case of severe and recurrent symptoms of MCA, the diagnosis of MCAS need to be considered. To call a condition MCAS, the following consensus criteria defined by Valent et al. [3,4], are required to be fulfilled:

1. typical clinical features of severe, acute systemic MCA (especially signs and symptoms of anaphylaxis);
2. increase in serum total tryptase level by at least 20% above baseline plus 2 ng/mL during or within four hours after a symptomatic period;
3. response of symptoms to MC blocking agents. MCA symptoms may range from mild to severe and sometimes life-threatening, correlating with the extent of mediator release from MCs [4].

Common symptoms of systemic MCA are acute urticaria, flushing, abdominal cramping, diarrhea, tachycardia, hypotension, and syncope [1,2,3,4]. Acute, severe, and recurrent symptoms involving more than one organ or tissue often with severe hypotension and anaphylaxis are generally present in MCAS. It is important to point out that MCA may present with chronic and less severe, nonspecific symptoms; however these symptoms alone are not considered sufficient criteria for MCAS [1,2,3,4]. Of note, MCAS may occur not only in the context of SM.

MCAS is sub-divided in primary or clonal MCAS in which a clonal MC disease is identified, the KIT D816V mutation is detected and MCs aberrantly express CD25 in most cases; secondary MCAS where an IgE-mediated allergy or another hypersensitivity/immunologic reaction that can induce MCA is diagnosed, in absence of neoplastic MCs or KIT D816V mutation. Idiopathic MCAS is diagnosed when the consensus criteria for MCAS are fulfilled, but no neoplastic MCs, no IgE-dependent allergy, no other hypersensitivity reaction or immunologic disease are identified [3,4].

### 2.3. 2017 WHO Classification of Mastocytosis

In the current 2017 WHO classification, mastocytosis represents a separate category, not included any more among myeloproliferative neoplasms, and sub-divided into three main groups: a pure cutaneous mastocytosis (CM) with skin-limited disease, more frequent in children and with good prognosis; SM characterized by involvement of one or more extra-cutaneous organs, more common in adults and with less favorable outcome and mast cell sarcoma (MCS), a very rare, localized and high-grade MC tumor [5] (Table 1).

In all patients with clinical suspicion of SM, the BM biopsy represents the first diagnostic step.

### 2.4. 2017 WHO Diagnostic Criteria of Mastocytosis

According to the current WHO classification, the diagnosis of SM can be made when the major criterion and one minor criterion or at least three minor criteria are met (Table 2) [5].

The major criterion is represented by multifocal, dense aggregates, each of at least 15 MCs, in BM biopsy and/or another extra-cutaneous organ.

The minor criteria are the following: (1) >25% of atypical or spindle-shaped or immature MCs in BM biopsy or BM aspirate or other extra-cutaneous organ; (2) abnormal MC phenotype (CD25 positivity with or without CD2 expression, being CD25 the most sensitive marker of neoplastic MCs); (3) the presence of an activating mutation at codon 816 of KIT gene in BM, blood or other extra-cutaneous organ; (4) persistently elevated serum tryptase (>20 ng/mL), in absence of an associated myeloid neoplasm [5].

### 2.5. “B” and “C” Findings and 2017 WHO Classification of Mastocytosis

Signs of an excessive MC burden in the tissues, called “B” findings, and signs of MC-related organ damage, called C findings (where C stands for requiring cytoreductive therapy) are used to further sub-classify SM [5].

Based on “B” findings (>30% BM infiltration and tryptase level > 200 ng/mL; signs of dysplasia or myeloproliferation in non-mast cell lineages, insufficient for diagnosis of an associated hematological neoplasm with normal or only slightly abnormal blood counts; abnormal size of lymph nodes, liver and spleen without impaired function) and on “C” findings (cytopenia due to massive BM infiltration; hepatomegaly with impaired liver function; splenomegaly with hypersplenism; malabsorption and weight loss for GI MC infiltrates; osteolytic bone lesions), SM is sub-divided into indolent SM (ISM; no B or C findings), smoldering SM (SSM; at least 2 B findings, but no C findings), and aggressive SM (ASM; at least one C finding) [5]. Two other categories of SM are SM with an associated hematological neoplasm (SM-AHN) and mast cell leukemia (MCL) [5] (Table 2). The term advanced SM is used for ASM, SM-AHN, and MCL.

In patients affected by SM-AHN, the clinical course of the disease depends both on systemic mastocytosis and on the associated hematological neoplasm [5], although in many cases the prognosis is determined mainly by the associated hematological neoplasm [5]. Any myeloid, lymphoid, or plasma cell neoplasms may be associated to SM, although myeloid neoplasms are the most frequently associated hematological neoplasms and plasma cell dyscrasia the least commonly associated disease [14]. Among SM, MCL is the rarest form (<1% of SM) and follows the worst prognosis [5]. To establish a diagnosis of MCL, the WHO criteria of SM need to be fulfilled and the presence of at least 20% of immature MCs in BM aspirate is required [5]. MCL is divided into the leukemic variant with at least 10% of MCs in the peripheral blood (PB) and the aleukemic variant (less than 10% of MCs in PB), which seems to be the more common of the two variants [5]. MCL is also divided into a more common acute form, with organ damage (C findings) and a rare chronic form, without organ damage [5].

### 2.6. Symptoms and Signs in Systemic Mastocytosis

Clinical manifestations in systemic mastocytosis have been divided into four groups: constitutional symptoms (asthenia, weight loss, fever); skin symptoms (flushing, pruritus, urticaria); systemic symptoms related to mediator release (tachycardia, hypotension, gastrointestinal symptoms, respiratory symptoms); and musculoskeletal symptoms (bone pain, myalgias, osteoporosis and fractures).

Organomegaly such as hepatomegaly, splenomegaly, and lymphadenopathy is absent in ISM, whereas it is usually present in SSM, which, by definition, is characterized by at least 2 B findings (organ involvement without organ dysfunction).

Symptoms related to organ impairment (C findings) can occur in ASM [5]. In SM, peripheral blood evaluation may reveal leukocytosis with eosinophilia, anaemia, neutropenia, and thrombocytopenia. BM impairment is present only in ASM or MCL [5].

### 2.7. Gastrointestinal Symptomatology in Systemic Mastocytosis

GI symptomatology represents one of the major causes of complaint in patients with mastocytosis [6,7,8,9] and gastroenterologists are among the specialists dealing with this complex disease. Sokol et al. compared GI manifestations of a large cohort of patients affected by mastocytosis with matched healthy subjects [15]. The authors concluded that GI manifestations in patients with mastocytosis are highly prevalent and often severe [15]. Many patients, even in absence of apparent GI involvement, show significant GI symptoms, which are mainly the result of mediator release causing hypermotility, bloating, diarrhea, abdominal cramping, nausea, and vomiting. Symptoms may also be due to direct infiltration of the gut mucosa by neoplastic MCs, leading to malabsorption and weight loss [11,13,15].

The rates of GI symptoms in mastocytosis have varied considerably, but recent studies reported GI clinical manifestations in 60–80% of SM patients [6,7,8,9].

Diarrhea and bloating are the most commonly reported GI symptoms, followed by nausea and abdominal pain [15]. Vomiting is reported in 10% of cases [15].

The mechanism leading to SM-related diarrhea is not completely elucidated, although Sokol et al. noted that non-D816V KIT mutations appeared more often associated with diarrhea than the D816V-mutated counterpart [14,15]. In their study, the authors supposed that, in absence of D816V KIT mutation, but in presence of other KIT mutations, such as exon 9 or 10 mutations, or wild type KIT, the disease could be locally more invasive, causing diarrhea. This feature was also noted in patients with MCL without D816V KIT mutation [15,16].

The sign of GIT damage due to MC infiltration is represented by malabsorption, in 5–30% of SM, with consequent weight loss [8,9,11]. Of note, GI mucosal involvement by mastocytosis without malabsorption and weight loss does not seem to correlate with aggressive disease [13].

Patients with mastocytosis are at more risk of developing peptic ulcer, possibly due to high levels of histamine, resulting in gastric acid hypersecretion [7,15,17,18]. Patients with MCL have a high frequency of gastroduodenal ulcers; in MCL, there is a massive MC tissue infiltration leading to high histamine secretion with consequent ulcer formation [15,19].

According to the largest MCL study by Georgin-Lavialle et al. [19], GI manifestations are among the most frequent complaints in MCL patients, with diarrhea (28%), anorexia (20%), severe weight loss of >10% of total body weight (38%), and GI ulcers (29%) often complicated by gastrointestinal hemorrhage (64%). Interestingly no gastroduodenal ulcers were present in patients with secondary MCL, evolving from SM, whereas they were identified in 38% of de novo MCL patients. As gastroduodenal ulcers are related to histamine release by neoplastic MCs, Georgin-Lavialle et al. suggested that in de novo MCL, neoplastic MCs might proliferate rapidly, infiltrating different organs, including the GIT, and degranulate with release of large amount of histamine leading to gastroduodenal ulcer [19]. Liver involvement can be observed in ASM with elevated liver enzymes, portal hypertension, and ascites [11].

Hepatomegaly and splenomegaly are the most frequent clinical signs, present in 68% and 65% of MCL patients, respectively [19]. Ascites and portal hypertension are seen in 18 and 16% of cases, respectively [19]. Rarely, SM has been reported as a cause of colonic ulcers closely mimicking Crohn’s disease [20].

### 2.8. Gastrointestinal Involvement by Systemic Mastocytosis: Endoscopic and Histological Features

Many patients with mastocytosis do not undergo endoscopic GI biopsies, therefore the true frequency of GI involvement in patients suffering from GI symptoms is not well defined [13].

The major diagnostic criterion for SM is the presence of MC aggregates (defined as 15 or more MCs) in BM or extracutaneous organs, including the GIT [5]. When SM is suspected, the diagnosis is generally made on BM biopsy, although GI biopsies could be an alternative method to identify SM in some patients [13].

In the study by Doyle et al., 66% GI mucosal biopsies were involved by mastocytosis, with the sites involved being ileum (86%), colon (81%), duodenum (67%), and stomach (35%) [13].

In 30–40% of cases, no remarkable endoscopic abnormalities are identified; in about 60% of cases, nonspecific endoscopic abnormalities including mucosal nodularity, erythema, erosions, or loss of mucosal folds are reported [13,15,21].

The diagnosis of mastocytosis in GI mucosal biopsies can be challenging as MC infiltrate can be focal and subtle. Multiple biopsies are necessary as insufficient sampling may miss the infiltrate [13,15,21].

**Figure 1:**
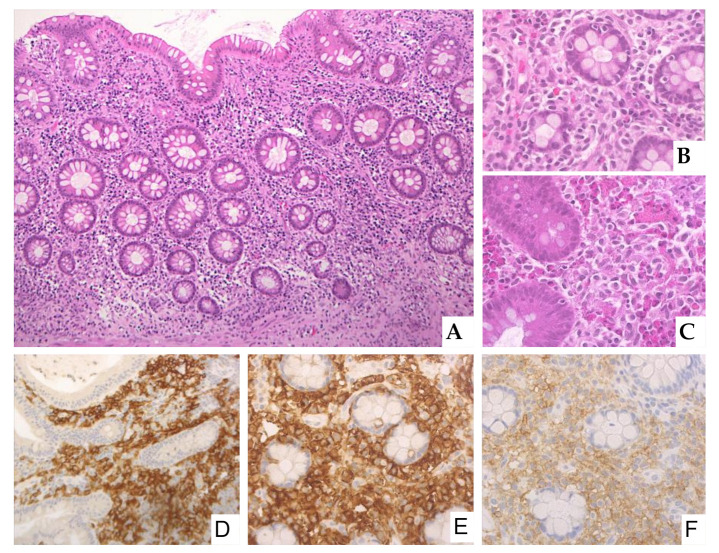
Low power view of large bowel lamina propria expanded by a polymorphous infiltrate of oval to spindle-shaped mast cells with admixed granulocytes, small lymphocytes, and plasma cells (panel (**A**), Hematoxilin and eosin, magnification 100×). High power view of the infiltrate in the lamina propria (panel (**B**), Hematoxylin and eosin, magnification 400×). High power view of the infiltrate rich in eosinophilic granulocytes (panel (**C**), Hematoxylin and eosin, magnification 400×). Tryptase positivity highlighting mast cell infiltrate (panel (**D**), immunostain, magnification 400×); CD117 positivity (panel (**E**), immunostain, magnification 400×); CD25 expression indicative of clonal mast cells (panel (**F**), immunostain, magnification 400×).

MCs infiltrate is composed of tiny clusters of cells, with a predominant sub-epithelial distribution [13], ranging in shape from rounded cells with moderate amount of pale, eosinophilic cytoplasm to ovoid and spindle-shaped cells with scant cytoplasm (Figure 1) [13,15].

A dense eosinophil infiltrate associated to MCs may sometimes obscure MCs, leading to the erroneous diagnosis of eosinophilic colitis [6,13]. In mastocytosis involving the GIT, the subtle MC infiltrate, sometimes even overwhelmed by eosinophils, may be difficult to identify unless special stains such as tryptase, CD117, and CD25 are performed [6,13,15,21]. MCs, both in neoplastic and reactive conditions, express CD117 and tryptase. CD117 is sensitive, but not specific for MCs; tryptase is less sensitive, but more specific. Both CD117 and tryptase are not diagnostic of the neoplastic nature, whereas CD25 expression is considered a hallmark of “MC atypia”. CD2 is less useful than CD25, being not always expressed in neoplastic MCs [5]. Aberrant CD30 expression is observed mainly in advanced SM and MCL, although rare cases of ISM may express CD30 as well as rare advanced SM may lack CD30 expression [5]. Doyle et al. did not find an association between CD30 expression by neoplastic MCs in the GIT and aggressive disease [13]. Of note, MCs in the GIT are reported to have a variable tryptase expression, which can lead to missing the infiltrate [13,15,21]. MCs can even be misinterpreted as hystiocytes due to the expression of CD68PGM1, a marker traditionally expressed by hystiocytes [6,15,21].

### 2.9. Diagnostic Issues

In patients with isolated or predominant GI symptoms, the identification of mastocytosis can be very difficult as symptoms overlap with many other more common GI diseases such as gastroesophageal reflux, *Helicobacter pylori* gastritis, peptic ulcer, inflammatory bowel syndrome (IBS), inflammatory bowel disease (IBD), celiac disease, and even carcinoma, which need to be ruled out.

The clinical work up of patients with GI symptoms suspected of having SM begins from serum tryptase evaluation. An elevated serum tryptase level above 20 ng/mL is a typical finding of SM and further exams including BM biopsy and KIT mutation analysis are then recommended.

In the study by Sperr et al. reviewing 43 SM cases, elevated serum tryptase level was found in the vast majority of patients with a high correlation between tryptase level and grade of BM infiltration by MCs [22]. However, a normal tryptase level does not exclude a MC disorder and therefore, in the suspect of mastocytosis, blood KIT testing and BM biopsy are considered essential.

Histologically, in the GIT, the main differential diagnosis of mastocytosis is with normal mucosa, the MC infiltrate being often very subtle and easily missed without the appropriate immunostains.

Additionally, mastocytosis need to be differentiated from eosinophil-rich diseases as in GI mastocytosis, MCs can be obscured by a prominent eosinophilic component [13,15,21]. The histological pattern of eosinophilic colitis can be associated with several causes, among which, IBD, drug injury, food allergy, and parasitic infections are the most common [23,24,25]. In parasitic infections as well as in IBD, MCs may be slightly increased, but they do not form aggregates and do not express CD25.

An interesting and not well clarified issue, arisen by Johncilla et al., regards abnormal MC aggregates incidentally found in GI mucosal biopsies of patients without suspected or established SM [26]. According to Johncilla et al., when MC aggregates occur in patients without a history of SM and minimal or mild GI symptoms, they may not reflect generalized disease and in the pathology report, the use of a descriptive term such as atypical enterocolic MC aggregates might be preferable, before complete patient’s work-up [26].

Additionally, up to 4% of the general population presents a recently described autosomal dominant-inherited genetic feature called hereditary alpha-tryptasemia (HαT) [27,28]. Individuals with this genetic trait have extra copies of the MC tryptase gene TPSAB1 encoding for serum tryptase that result in high baseline serum tryptase level > 8 ng/mL. Clinical symptoms in HαT are variable, including flushing, urticaria, and GI manifestations, therefore simulating MC disorders [28].

Interestingly, in this setting of individuals, Hamilton et al. recently found an increase in duodenal MCs, usually more than 20 MCs/HPF, in clusters < 15 MCs, but without signs of clonality [29].

### 2.10. Molecular Features

More than 90% of patients harbor a gain of function KIT mutation and more than 80% have the KITD816V mutation [5,30,31]. The degree of KIT D816V mutational status varies according to the different degree of MC tissue infiltration [32]. Peripheral blood KIT testing can be used to help establishing the clonality of MC disease [33]. Unlike other types of SM, only 40–67% of MCL cases have the typical KIT D816V mutation [5,34,35]. In a proportion of MCL cases, atypical mutations in the KIT gene such as D816H/Y or F522C are identified or wild-type KIT may be present [5,34,35]. In ASM and MCL, concurrent mutations in SRSF2, TET2, ASXL1, RUNX1, K/N-RAS, CBL, and EZH2 can be seen [5,34,35,36].

The presence of SRSF2/ASXL1/RUNX1 mutations (S/A/R) in patients with MCL has been proposed to be an independent predictor for poor survival [5,34,35,36].

### 2.11. Treatment

Treatments of SM are of two types: medications to control MC mediator-related symptoms and cytoreductive treatments to limit MC burden and increase survival mainly in ASM and MCL. In ISM and SSM, the management is mainly symptomatic plus a correct monitoring to identify signs of progression. Additionally, all SM patients should be aware of potential triggers of MC activation. The triggers of MC degranulation may be different, including emotional stress, physical stimuli, infections, allergies, and drugs such as alcohol, aspirin, non-steroid anti-inflammatory drugs, and opioids.

Treatments of MC mediator GI symptoms rely on anti-mediator therapy such as antihistamines [12]. H_1_ and H_2_ receptor antagonists are often used in combination. H_1_ antagonists can treat skin symptoms [22]. To control GI symptoms, H2 antagonists are generally used as 1st-line therapy and proton pump inhibitors as 2nd-line [37,38]. Cromolyn sodium as a MC stabilizer has been reported to be effective in reducing GI symptoms (especially diarrhea, abdominal pain, and nausea) and it is used as 3rd-line therapy [39]. Leukotriene antagonists and IgE-binding treatment with the monoclonal antibody omalizumab are included among therapies to treat MC activation symptoms [40,41]. The use of systemic steroids is limited to specific situations, for instance in the setting of organ damage; low-dose corticosteroids are reported to reduce malabsorption and ascites [42].

In ASM, treatments interfering with MC proliferation and survival are used. Tyrosine kinase inhibitors (TKIs), chemotherapy, and allogenic stem cell transplantation (allo-SCT) are considered the best therapeutic options for ASM, including MCL [43,44,45]. Imatinib and Nilotinib demonstrate effect against wild-type KIT and a limited activity against KIT D816V; however, there are reports of response to these drugs in patients with atypical KIT mutations [5,46]. Midostaurin, a TKI with activity against KIT D816V, represents the only approved therapy for advanced SM, including MCL [47]. In advanced SM, most responses with Midostaurin are only partial and not sustained. Other medications potentially effective for SM patients are under investigation. Recent and promising results have been obtained with Avapritinib, a KIT and PDGFRA inhibitor [48,49]. Interferon alpha and cladribrine have also been used [50,51]. Allo-SCT needs to be considered in young and otherwise healthy patients with ASM, as this is the only option for a sustained response [52]. Prior to SCT, therapies such as either midostaurin or cladribrine should be used to bring the patients to the best response.

## 3. Discussion

MCs are important components of the immune system, inducing a protective response of the host against pathogens, particularly at the mucosal border between our body and the external environment.

Moreover, MCs are components of the inflammatory microenvironment modulating carcinogenesis [53,54]. Depending on the milieu, MCs represent a source of either pro-tumorigenic factors favoring angiogenesis and tumor growth or anti-tumorigenic molecules (TNFα and IL-9) with a protective function. The role of MCs and their mediators seems to be cancer specific as MCs have a pro-tumorigenic role, for instance, in colorectal cancer or a protective role as in breast cancer. In other neoplasms, MCs are apparently innocent bystander cells [55,56] Therefore, MCs could represent potential therapeutic targets in tumors [55,56]. More studies are essential to completely understand the role of MCs in cancers.

On the other hand, while the association of mastocytosis with other hematological tumors is well known and encompassed by the category of SM-AHN [5,14,57], the risk of solid cancers in patients with SM remains controversial [58,59].

Mastocytosis is often a disease hard to diagnose, in particular the correct identification of an underlying MC disorder is difficult, especially in patients with isolated GI symptoms. Clinical symptoms are nonspecific and overlap with symptoms of other more common GI diseases.

In the clinical suspect of SM, most patients undergo BM biopsy; however, endoscopic GI biopsies may represent a valid alternative to establish the diagnosis of SM in selected cases, particularly in patients with GI symptoms.

To date, the true frequency of GI involvement by mastocytosis in patients suffering from GI symptoms remains not well defined, as many patients do not undergo endoscopic biopsies [13].

In SM involving the GIT, mucosal nodularity represents a common endoscopic appearance [13]. However, as in 40% of cases with GI involvement, no endoscopic abnormalities are present and MC infiltrate can be focal and subtle, an adequate sampling with multiple biopsies is essential in order not to miss the infiltrate [13,15,21]. Tryptase represents the most specific MC marker; however, in the GIT, tryptase can show a variable expression and it can be positive only in a limited number of cells, representing a potential diagnostic pitfall [13]. The variable tryptase expression may be related to differences in the composition of MC granules in the GIT [13]. Therefore, CD117 needs to be performed to identify MCs in GIT. The expression of CD25 by MCs is considered indicative of their clonal nature [11] and, in the correct clinical context, confirms the diagnosis of SM.

MCs are normally resident cells in the gut and are found as isolated elements in the gut of healthy subjects. To date, there is no agreed value for what has to be considered an increase in MCs in intestinal mucosa and which might be the clinical significance of an increase in normal-appearing, sparse MCs [29].

In patients with diarrhea-predominant IBS, an increase of singly dispersed, not cluster-forming MCs, has been reported in some studies [60,61], but not supported by other studies [8,13,62].

Recently Hamilton et al. found an increase of MCs (>20 MCs/HPF, in clusters < 15 cells) in the duodenal mucosa of a cohort of individuals with HαT and symptoms of MC activation [29]. The increase in MCs might contribute to the GI symptoms usually present in individuals with this genetic trait [29]. The authors suggested to perform appropriate stains to identify MCs in GI biopsies obtained from patients with MC activation symptoms, including GI manifestations, and with a baseline serum tryptase > 8 ng/mL; additionally, genetic testing for HαT is advisable [29]. Unlike SM, the increase of MCs in GI biopsies of patients with HαT is not associated with clonal expansion [29].

Of note, the recent study by Johncilla et al., uncovered the complex issue of MC aggregates incidentally discovered in intestinal biopsies of asymptomatic patients or with only mild GI symptoms [26]. These aggregates were made up of 15 or more MCs, which were CD25 positive and, therefore, highly suggestive of SM, although no other features of SM were found in these patients [26]. This study underlines the importance of a complete patient’s work-up if aggregates of atypical MCs are observed in GI mucosa biopsies.

## 4. Conclusions

In SM, the GIT is frequently affected with rather vague and nonspecific clinical manifestations, making the diagnosis of GI mastocytosis particularly difficult.

In patients presenting with GI symptoms, which cannot be attributed to more common GI diseases, SM needs to be taken in consideration. Because endoscopy is often unremarkable and involvement can be focal, multiple biopsies should be taken by endoscopists. A high index of suspicion of both clinicians and pathologists is essential to correctly recognize GI involvement by mastocytosis.

## Figures and Tables

**Table 1:** Classification of mastocytosis.

**Cutaneous mastocytosis**
Urticaria pigmentosa/maculopapular cutaneous mastocytosis Diffuse cutaneous mastocytosis Solitary mastocytoma of the skin
**Systemic mastocytosis** (SM)
Indolent systemic mastocytosis (ISM) Meets criteria for SM. No B or C findings. No evidence of AHN **Smoldering systemic mastocytosis** (SSM) Meets criteria for SM; at least 2 B findings, but no C findings. No evidence of AHN **Systemic mastocytosis with an associated hematological neoplasm** (SM-AHN) Meets criteria for SM and criteria for an associated hematological neoplasm as a distinct entity according to WHO classification **Aggressive systemic mastocytosis** (ASM) Meets criteria for SM. One or more C findings **Mast cell leukemia** (MCL) Meets criteria for SM plus at least 20% of immature MCs in BM aspirate; MCL can be leukemic (at least 10% of MCs in PB) and aleukemic (less than 10% of MCs in PB). MCL is also divided into an acute form, with organ damage (C findings) and a chronic form, without organ damage
**Mast cell sarcoma** Unifocal mast cell tumor. No evidence of SM. Destructive growth pattern. High grade cytology

Legend: AHN: associated hematological neoplasm; ASM: aggressive systemic mastocytosis; BM: bone marrow; ISM: indolent systemic mastocytosis; MCs: mast cells; MCL: mast cell leukemia; PB: peripheral blood; SM: systemic mastocytosis; SM-AHN: systemic mastocytosis with an associated hematological neoplasm; SSM: smoldering systemic mastocytosis; WHO: World Health Organization.

**Table 2:** WHO diagnostic criteria for systemic mastocytosis.

The diagnosis of SM can be made when the **major criterion and one minor criterion** *or* at least **3 minor criteria** are fulfilled
**Major criterion**
Multifocal and dense MCs infiltrates (= or >15 MCs in aggregates) in BM sections and/or other extracutaneous organ
**Minor criteria**
>25% of MCs are spindle-shaped or atypical in biopsies of BM or other extracutaneous organs or >25% of MCs in BM aspirate smears are immature or atypical detection of an activating point mutation at codon 816 of KIT in BM, blood or other extracutaneous organ MCs in BM, blood or other extracutaneous organ expressing CD25 with or without CD2 (in addition to normal MC markers) Persistently elevated serum tryptase level (>20 ng/mL), unless there is an associated myeloid neoplasm in which case this parameter is not valid
**“B” findings**
High MC burden in BM biopsy: >30% of cellularity composed by MCs (focal, dense aggregates) and serum total tryptase level >200 ng/mL Signs of dysplasia or myeloproliferation in non-mast cell lineage(s), but insufficient criteria for AHN diagnosis, with normal or only slightly abnormal blood counts Hepatomegaly (without impairment of liver function); palpable splenomegaly (without hypersplenism) and/or lymphadenopathy
**“C” findings**
BM dysfunction due to MC infiltration, manifested by cytopenia in 1 or more lineages (ANC < 1.0 × 10^9^/L: Hb < 10g/L and/or PTL count < 100 × 10^9^/L Palpable hepatomegaly with impairment of liver function, ascites, or portal hypertension Skeletal involvement with large osteolytic lesions with/without pathological fractures (pathological fractures caused by osteoporosis do not qualify as a “C” finding) Palpable splenomegaly with hypersplenism Malabsorption with weight loss due to gastrointestinal MC infiltrates

Legend: AHN: associated hematological neoplasm; ANC: absolute neutrophil count; BM: bone marrow; MC: mast cell; MCs: mast cells; PTL: platelet; SM: systemic mastocytosis; WHO: World Health Organization.

## Data Availability

Individual patient data from the original studies included in the present review is not available and data sharing at this level is not applicable for a systematic review.
